# The Luminescence of 1,8-Diazafluoren-9-One/Titanium Dioxide Composite Thin Films for Optical Application

**DOI:** 10.3390/ma13133014

**Published:** 2020-07-06

**Authors:** Aneta Lewkowicz, Robert Bogdanowicz, Piotr Bojarski, Mattia Pierpaoli, Ignacy Gryczyński, Anna Synak, Michał Mońka, Jakub Karczewski, Wiktoria Struck-Lewicka, Renata Wawrzyniak, Michał J. Markuszewski

**Affiliations:** 1 Faculty of Mathematics, Physics and Informatics, University of Gdańsk, Wita Stwosza 57, 80-308 Gdańsk, Poland; piotr.bojarski@ug.edu.pl (P.B.); anna.synak@ug.edu.pl (A.S.); michal.monka@ug.edu.pl (M.M.); 2 Faculty of Electronics, Telecommunications and Informatics, Gdańsk University of Technology, 11/12 Gabriela Narutowicza Street, 80-233 Gdańsk, Poland; rbogdan@eti.pg.edu.pl (R.B.); mattia.pierpaoli@pg.edu.pl (M.P.); 3 Faculty of Microbiology, Immunology and Genetics, Health Science Center, University of North Texas, 3500 Camp Bowie Boulevard, Fort Worth, TX 76107, USA; Ignacy.Gryczynski@unthsc.edu; 4 Faculty of Applied Physics, Gdańsk University of Technology 11/12 Gabriela Narutowicza Street, 80-233 Gdańsk, Poland; jakub.karczewski@pg.edu.pl; 5 Department of Biopharmaceutics and Pharmacodynamics, Medical University of Gdańsk, Al. Gen. Hallera 107, 80-416 Gdańsk, Poland; wiktoria.struck-lewicka@gumed.edu.pl (W.S.-L.); renata.wawrzyniak@gumed.edu.pl (R.W.); michal.markuszewski@gumed.edu.pl (M.J.M.)

**Keywords:** DFO, TiO_2_, thin films

## Abstract

The investigation of innovative label-free α-amino acids detection methods represents a crucial step for the early diagnosis of several diseases. While 1,8-diazafluoren-9-one (DFO) is known in forensic application because of the fluorescent products by reacting with the amino acids present in the papillary exudate, its application for diagnostic purposes has not been fully investigated. The stabilization of DFO over a transparent substrate allows its complexation with biomolecules for the detection of α-amino acids. In this study, DFO was immobilized into a titanium dioxide (TiO_2_) matrix for the fluorescence detection of glycine, as a target α-amino acid (a potential marker of the urogenital tract cancers). The DFO/TiO_2_ composite was characterized by atomic force microscopy, spectroscopic ellipsometry, fluorescence spectroscopy and fluorescence microscopy. The performed fluorescent studies indicate spectacular formation of aggregates at higher concentration. The measurements performed using various fluorescence and microscopic techniques together with the suitable analysis show that the aggregates are able to emit short-lived fluorescence.

## 1. Introduction

The aromatic ketone, 1,8-diazafluoren-9-one (DFO) contains heteroatoms (nitrogen and oxygen) in a ring structure. In our previous study, we investigated the solvation effects and the influence on the lowest singlet excited state of DFO. It was shown that, in protic environments, DFO forms a solute–solvent hydrogen bond complex in its ground and excited state [1].

Research carried out in this work shows in turn the concentration-dependent properties of DFO in TiO_2_ matrix, which can be attributed to the formation of aggregates in pores of the examined matrix, where mean local concentration of the DFO is considerably higher than that in bulk solution. The presence of DFO aggregates, in concentrated systems, may significantly influence the physicochemical and biological processes after the photoexcitation of the system. Therefore, one of the goals of this work was to study basic aggregate properties in the titanium dioxide thin film, which is important for its potential application, since the aggregation process can shorten the fluorescence decays and lifetimes of monomers. This is the case of many other molecular systems in which aggregates are formed [2,3,4,5,6], for example in some medical applications [7,8]. The knowledge of whether we are dealing with aggregate or monomer form at a given concentration of the active compound is vital as the actual form may alter the pharmacological activity of the compound as well as its ability to penetrate through biological membranes. Molecular aggregates also play a crucial role as energy transmitters from sunlight in many biological systems [9,10,11,12] and their unusual optical properties are frequently studied and used in various fields of nanoscience and nanotechnology such as efficient energy collecting, spectral broadening of the optical response of fluorescent systems and biosensing on plasmonic platforms [13,14,15]. Although, TiO_2_ nano-objects have demonstrated toxicity and ecotoxicity [16,17,18,19], its use in the biomedical devices is widespread and in many cases does not seem to lead to serious side effects. The affinity of DFO with TiO_2_ and the versatility of the sol–gel technique allow designing and developing an organic–inorganic framework exhibiting the desired optical/chemical properties. In particular, the transparency over a broad spectral range allows the use of spectroscopic techniques for the investigation of chemically-bonded molecules and the intermolecular processes occurring in the matrix to be investigated. Moreover, the facile preparation allows the substrate properties to be tailored, such as the pore size and density, to create materials with spatially different distributions of fluorophores, which, in turn, can tune the desired optical properties [20,21]. This study aimed to propose a potential luminescent probe in the form of a thin TiO_2_ film with an integrated DFO dye and to optimize the spectroscopic features of the luminescent probe, which is very important in the selection of cancer markers.

Table 1 presents the exemplary, recently proposed metabolic markers of these diseases [22]. However, it should be underlined that highly specific and sensitive indicators have not been confirmed and validated in clinical practice yet.

Recently, the metabolomics approach has been commonly applied to discover and propose new, specific metabolic indicators of urogenital tract cancer disorders, especially prostate cancer [22,23,24]. The VIP (variable importance into projection) scores were computed and variables with a VIP score of >1 were considered important in this model; glycine is proposed as a potential biomarker of urogenital tract cancer. The elaboration of a sensitive composite for the estimation of glycine seems important from the point of view of further potential applications, i.e. a luminescent probe for potential biomarkers. The ability of DFO to form complexes with specific α-amino acids (e.g., glycine, sarcosine, etc.) and the idea of using this compound, after introducing it into the TiO_2_ matrix, as a potential marker of the metabolic urogenital carcinoma was the reason for the study of spectroscopic properties of DFO presented in this work.

To the best of our knowledge, this is the first attempt to study the spectroscopic properties of potential metabolic markers of urogenital tract cancer incorporated into a thin layer matrix of TiO_2_ and characterized as a biomaterial.

## 2. Materials and Methods

All reagents used in this study were of analytical grade. 1,8-diazafluoren-9-one was purchased from Aldrich (Sigma-Aldrich Munich, Germany) and it was spectroscopically pure (dye content 99%). Titanium(IV) tetra(2-propanolate)-99.000% trace metals basis, poly(ethylene glycol) p-(1,1,3,3-tetramethylbutyl)-phenyl ether (Triton X-100), hydrochloric acid, propan-2-ol and pentane-2,4-dione were purchased from Aldrich (Sigma-Aldrich, Munich, Germany). Ethanol was purchased from POCH Company (POCH Company, Gliwice, Poland). Deionized water was obtained from a Hydrolab system, prior to use. The sol–gel method was adopted to prepare DFO/TiO_2_ thin films. The DFO/TiO_2_ precursor solution was obtained using titanium(IV) tetra(2-propanolate), propan-2-ol, Triton X-100 and hydrochloric acid (37%). Separately, DFO was dissolved in ethanol. Next, both solutions were mixed by vigorous stirring. Three drops of sodium hydroxide (10^−1^ [M]) were used as a catalyst to promote the reaction of the sol–gel process. The so-obtained solution was distributed over a clean piece of a microscopic glass using the spin-coating technique (SCI-40 LOT, Oriel spin coater) at 60 rpm for 60 s, in order to obtain a homogenous thin film. The sol–gel process allows DFO to be incorporated into TiO_2_ matrices at room temperature and atmospheric pressure. Using this method, the following concentrations of the dye were obtained: 2 × 10^−2^, 1 × 10^−2^, 2 × 10^−3^, 1 × 10^−3^, 1 × 10^−4^ and 1 × ^10−5^ [M]. The optical density of the films was in any case below 0.1, which is low enough to neglect the inner filter effects. The thickness of the thin films was tuned by controlling the gelation times through the sol–gel method. The gelation time was measured from the moment of mixing all of the components and, for the purpose of this work, was kept at 110 min.

### Apparatus

The topography and the roughness of the surface were analyzed using atomic force microscopy (AFM Nanosurf Easyscan 2, Nanosurf, Liestal, Switzerland) in contact mode. The surface analysis of images was conducted using Gwyddion 2.47 software (Department of Nanometrology, Czech Metrology Institute, Brno, Czech Republic). Atomic force microscopy (AFM) images were collected by scanning dry sample wafers with an atomic force microscope equipped with an AFM dry scanner and APPNANO SICON probe which is nanofabricated using highly doped single crystal silicon. This probe has a long, thin cantilever allowing for a low spring constant (0.29 N/m). The AFM scanner was calibrated using a standard calibration grid as well as 100 nm diameter gold nanoparticles (T = 20 °C, the relative humidity = 60%, the atmosphere: air).

Spectroscopic ellipsometry (SE) studies were handled using a Jobin-Yvon UVISEL phase-modulated ellipsometer (HORIBA Jobin-Yvon Inc., Edison, Middlesex County, NJ, USA) over the 300–1100-nm wavelength range. The SE measurements were carried out at 60° angle of incidence in agreement with the Brewster’s angle of the quartz glass substrate. The DeltaPsi software (v. 2.4.3) (HORIBA, Kyoto, Japan) was used to determine the spectral variations of refractive index n(λ) and the extinction coefficient k(λ) of DFO/titanium dioxide composite films. Additionally, the optical band-gap was derived by means of a Tauc plot. The dispersion of nanocomposite DFO/TiO_2_ films was simulated by the Forouhi–Bloomer model [25], coherent with the Kramers–Kronig approach, relevant to the amorphous and polycrystalline TiO_2_ phase [26].

The fluorescence spectra and fluorescence intensity decays were measured upon front face excitation (magic angle mode) with a universal spectrofluorometer (laser LDH-D-C-470-Picoquant, Germany; photomultiplier-Hamamatsu H10721P-01, Hamamatsu Photonics K.K., Japan; monochromator- Shamrock 303i-B, Andor Technology, UK) constructed in our laboratory [10]. As an excitation source, we used an LDH380 laser emitting pulses of about 288 ps (FWHM) half-width at lambda = 380 nm (Pico-Quant, Germany).

To obtain two types of images of the samples, namely fluorescence (epi-illumination mode, U-FBW Fluorescence Filter Cube) and phase contrast, an inverted Olympus IX73 microscope (Olympus, Japan) was used. Both observations were performed with 10× objective (N.A. = 0.3, Olympus, Japan). The images were acquired using a monochromatic camera (Orca flash 4.0 CMOS, Hamamatsu, Japan) with the fixed exposure time t = 1 s.

Time-resolved emission spectra (TRES) were recorded using the pulsed spectrofluorometer (2501S Spectrograph, Bruker, Optics Inc., Billerica, MA, USA) described previously in detail [27].

## 3. Results and Discussion

### 3.1. Morphology of DFO/TiO_2_ Thin Films

**Figure 1:**
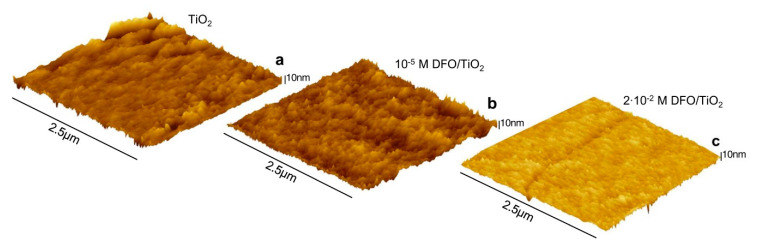
AFM image of thin films of: (**a**) TiO_2_; (**b**) 10^−5^M DFO/TiO_2_; and (**c**) 2·10^−2^ M DFO/TiO_2_.

The AFM topography images of DFO/TiO_2_ thin films with various DFO concentrations are displayed in Figure 1. The presented microscopic images are representative of the obtained samples. The thin film appears to be smooth with a roughness smaller than 1 nm for samples with DFO and slightly rougher (S_a_ > 2.5 nm) for pure TiO_2_. This result highlights the high smoothness of the prepared thin film, which suggests that DFO particles are rather evenly distributed in the TiO_2_ matrix. The presented structural analysis shows that the obtained hybrid thin films are homogeneous with low roughness, which decreases with increasing DFO concentration.

**Figure 2:**
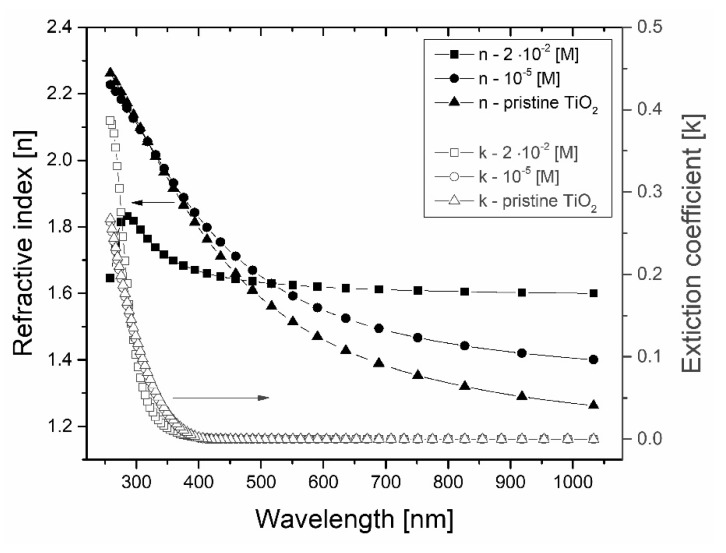
The variation of optical constants of DFO/TiO_2_ thin nanocomposite films as a function of DFO dye concentration.

Spectroscopic ellipsometry was utilized to investigate the optical constants and the thickness of the sol–gel deposited DFO/TiO_2_ nanocomposite thin films. Figure 2 illustrates the refractive index and extinction coefficient for TiO_2_-based nanocomposite films with various DFO dye concentrations. The strong relative variation of TiO_2_ optical constants was noticed once the DFO dye admixture was varied. Hence, the dispersion reveals normal behavior in the visible wavelength range as known for crystalline forms of TiO_2_.

The increasing DFO dye concentration induces slight increase of refractive index of TIO_2_ matrix from 1.51 to 1.59 at 550 nm. The largest observed refractive index reaches 1.62, which is attributed to the mixture of crystalline anatase and amorphous TiO_2_. It should be stated that the DFO/TiO_2_ thin nanocomposites were not annealed in the experimental procedure. Additionally, 10^−2^ M of DFO dye admixture causes a shift of resonance to the shorter wavelengths, as reported for amorphous or nanocrystalline structures [26].

The DFO induced variations in the refractive index (*n*@550 nm), energy bandgap, thickness and porosity *P* are listed in Table 2. The band gap *E_g_* of the films was indirect and was derived by use of the Tauc plot analysis.

The *E_g_* values decreases for increasing DFO concentration. Lower *E_g_* could be attributed to the shift in film density where the structure of the DFO/TiO_2_ thin films is converted from the amorphous (*E_g_* ~ 3 eV) phase to the denser nanocomposite form.

The porosity *P* of the DFO/TiO_2_ composites was approximated by the formula reported by Gartner et al. [28] for nanostructures. The relation between the refractive indices *n_@550nm_* of the studied DFO/TiO_2_ samples and the dense, non-porous anatase TiO_2_ phase (*n_d_* = 2.52) was used to estimate porosity rates (see Table 2).

Thereafter, the larger refractive indices (*n_@550nm_*) indicate the reduced porosity and increased film density caused by DFO incorporation. Such observations reveal homogenous incorporation of the DFO dye within the titania matrix decreasing also porosity. The densification effect is attributed to high polarity of DFO molecule stereochemistry during sol–gel process and improved crystallization of titania clusters. The fitting procedure gives accurate values of film thickness. The thickness of DFO/TiO_2_ thin films decreases with decreasing of concentration of DFO because of the elimination of ethanol and dyes residue. The minor increase of the film thickness is linked with the increased viscosity of the sol–gel precursors rich in DFO [29].

### 3.2. The Luminescence Properties of the DFO/TiO_2_ Thin Films

**Figure 3:**
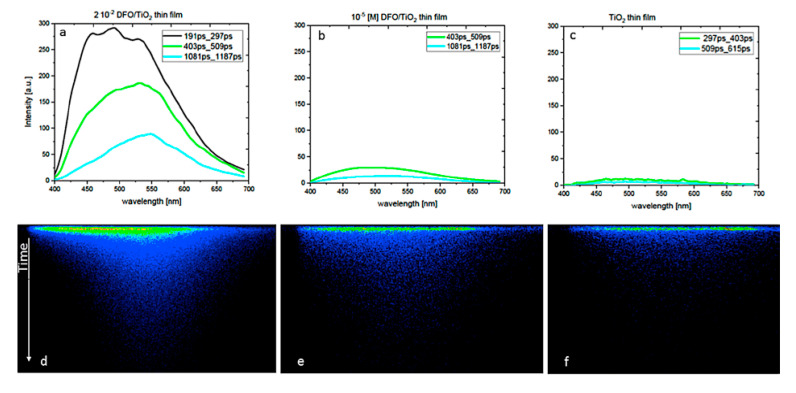
Time-resolved emission spectra of DFO/TiO_2_ thin films at several DFO concentrations ((**a**,**d**) 2 × 10^−2^ [M] DFO/TiO_2_; (**b**,**e**) 1 × 10^−5^ [M] DFO/TiO_2_; and (**c**,**f**) 0 [M]DFO/TiO_2_) at room temperature (T = 293 K). The excitation wavelength was 380 nm.

The luminescence properties of the DFO in TiO_2_ thin films were studied by fluorescence spectroscopy techniques. Figure 3 presents the time evolution of the fluorescence spectra for low and high concentrations of DFO in the TiO_2_ matrix and original TRES images, respectively.

The measurement results were obtained as a quasi-three-dimensional, colorful flat image, with the wavelength on the horizontal axis, the time on the vertical axis and the intensity of color scale. By slicing the image along the wavelength axis at a given time after the moment of excitation, the emission spectrum corresponding to that time was obtained. Experimental, trivial reasons for the changes observed such as inner filter effects were rejected by measuring the fluorescence at low optical densities, where such effects are negligible.

At the smallest DFO concentration (10^−5^ [M]) in TiO_2_ thin films, a fluorescence spectrum similar to the spectrum of TiO_2_ was observed. The matrix carries the contribution to sample fluorescence, but its intensity is smaller than that of the matrix with DFO.

Just after excitation, the emission spectrum of the sample with the highest DFO concentration consisted of three bands at 460, 500 and 550 nm. This means that we are dealing here with the monomer and aggregate emission. The time evolution of the emission spectrum shows that the monomer emission at 460 nm vanishes gradually and only the broad band with the maximum at 550 nm remains visible. Certain changes observed in the fluorescence spectra profiles with concentration may originate from the following reasons:

1. Emission of monomers and weakly fluorescent TiO_2_ matrix where both types of emitting species fluoresce in the similar spectral region. The emission close to about 470 nm was present in amorphous film TiO_2_, although with a small intensity [30]. An increase in the intensity of the monomer fluorescence signal at a small concentration of DFO generally indicates the influence of the TiO_2_ matrix. However, a decrease in the intensity of the monomer PL signal at a high concentration of DFO shows the creation of fluorescence aggregates, which are more dominant over the TiO_2_ matrix at long wavelengths.
2. DFO is a solvatochromically sensitive probe; therefore, under some changes in the matrix polarity, the effect of spectral shift could be anticipated. However, the matrix remains chemically unmodified for all the samples and the effect of a potential significant polarity change of the whole matrix by only a concentration increase of DFO molecules seems rather unlikely. The location of the fluorescence peak of DFO by comparison to the fluorescence of DFO and other fluorenone-like molecules in liquid solutions [1,31,32] suggests that the TiO_2_ matrix belongs to relatively nonpolar media. This is also in agreement with other results and discussions performed previously [25,26,33].

To get more insight into the photophysical properties of fluorescent species of DFO, the determination of mean fluorescence lifetimes from the measurements of fluorescence decays was made for different concentrations of DFO. Table 3 presents the results of the mean fluorescence lifetimes of DFO for different concentrations of the dye in the TiO_2_ matrix. Shortening of fluorescence lifetime with the increase in the dye concentration evidences the significant presence of aggregates, which can play a double role in the system: firstly, aggregates can act as perfect or imperfect traps for excitation energy transferred from monomers [2,12,34,35,36,37,38,39] and, secondly, the aggregates at highest concentrations are likely to contribute to the fluorescence signal emitting short living fluorescence. A similar behavior has been previously observed and analyzed for several other dyes such as rhodamines and carbocyanines in polymers and hybrid matrices with the only difference that, in this work, the fluorescence spectral shift was found more pronounced, making those analyses more straightforward [4,39].

All of the results support the hypothesis that the increase of the aggregation degree was induced by the hydrogen bonds (O-H^…^N). This was possibly due to the formation of intermolecular hydrogen bonds between the DFO and ethanol, water molecules or isopropanol molecules (the solvent remained in the pores of the TiO_2_ after the sol–gel process). As a result, the molecules could connect with neighboring molecules and form aggregates. Meanwhile, the aggregation extent increased with an increase of the concentration of DFO molecules in the thin films. These spectral changes were observed only in TiO_2_ thin films and may be attributed to the formation of aggregates, promoted by the TiO_2_ matrix.

### 3.3. Design of Luminescent Probe Sensitive to the Presence of the Markers of Urogenital Tract Cancer

The above information allowed us to design a luminescent probe sensitive to the presence of the proposed markers of urogenital tract cancer, i.e., α-amino acids [22]. The was the first attempt at reactivity studies of thin DFO/TiO_2_ films relative to glycine in a phosphate buffer.

**Figure 4:**
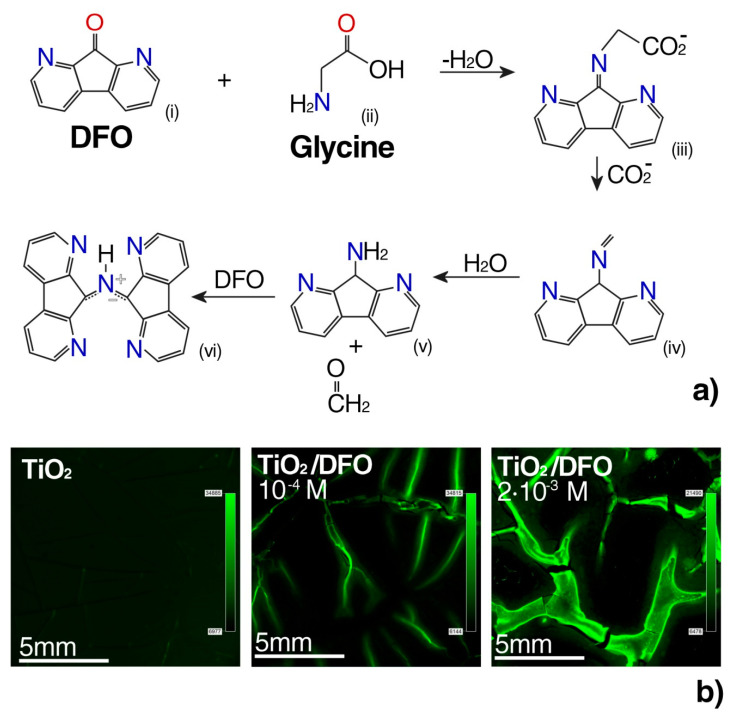
(**a**) Reaction of DFO with α-amino acids: The hemiketal attack from the nitrogen of amino acid at the electron-deficient carbon; after the loss of water, the imine is formed, which maintains the alkyl fragment from the amino acid and decarboxylation to form (IV). Hydrolysis shows at the nitrogen-carbon double bond formatting of an aromatic amine (V) and acetaldehyde and reacts with another DFO molecule to create 9-(1,8-diazafluoren-9-ylidene)amino-1,8-diazafluorenone (VI) [40]. (**b**) Fluorescence micrograph of DFO in TiO_2_ thin films at different DFO concentrations after reacting with glycine, with λ_exc_ = 450 nm.

The measurements performed using various fluorescence and microscopic techniques show that the aggregates are able to fluorescence. The concentration of DFO in the thin TiO_2_ film was optimized, in order to optimize the complexing reaction conditions of the DFO with the amino acids present in the phosphate buffer at pH = 7.4. Such conditions simulated the sample environment of urogenital tract cancer cells (Figure 4a).

The mechanism of formation of complex DFO with glycine was probed by reacting DFO with glycine (α-amino acids) in an ethanol solution. Glycine differs from other α-amino acids in ethanol to give a cycloadduct in which the carboxyl group was retained while the other α-amino acids underwent cycloaddition via the decarboxylated azomethine ylide [41].

In this study, biomaterials containing a complex luminophore-potential marker were studied using a fluorescence microscope. Figure 4 shows the fluorescent complex DFO with α-amino acids after impregnation in a buffer solution with glycine having a concentration of 10^−6^ [M] [42].

In Figure 4, we can observe that the fluorescence of complex fluorophores with biomarkers was induced by the high concentration of DFO in the TiO_2_ thin film. Negative control of the fluorescence micrograph without glycine (Figure S1) and the highest contrast in fluorescence for 2 × 10^−2^ [M] with glycine (Figure S2), have been reported in Supplementary Materials. For this reason, it is possible to control the aggregation degree of the dyes in the thin films, by varying the amount of DFO. These results suggest that it is possible to optimize the DFO aggregation within the matrix, exhibiting the desired luminescent properties, by a proper design of the DFO/TiO_2_ substrate matrix.

## 4. Conclusions

In this study, we observed enhanced solid-state luminescence by using TiO_2_ thin films in the presence of aggregates at high concentrations of DFO. We designed a luminescent probe, which can react with α-amino acids to create a fluorescent complex.

For the first time, it was found that the aggregation process of DFO, in the TiO_2_ matrix, at a sufficiently high concentration, developed rapidly. As confirmed by different complementary spectroscopic and microscopic techniques, the formed aggregates can emit short-lived fluorescence. The aggregation process is promoted both by the local surrounding of the DFO molecules to TiO_2_ nanochannels and pores and by the presence of residual solvents, which affect the ability of DFO to aggregate into larger structures. At high DFO concentration, aggregates were formed, which affect the fluorescence emission. We observed increased intensity of fluorescent with increasing concentration of DFO in TiO_2_ thin films (in short living fluorescence). Therefore, it seems reasonable to assume that the determined mean fluorescence lifetime of DFO at high concentration is connected to the presence also non-fluorescent aggregates of DFO.

In this work, we report that the synthesized DFO/TiO_2_ thin films can be successfully employed for the rapid detection of glycine, due to the enhanced fluorescence.

We believe that the results obtained will be helpful in further studies dedicated to biosensing platforms exploiting DFO as a luminescent probe to monitor non-fluorescent markers of urogenital tract cancer.

## Figures and Tables

**Table 1:** The list of putative metabolic markers of urogenital tract cancers obtained with the use of the metabolomics approach.

Metabolite	FDR *p* Value	VIP
Glycine	1.2 × 10^−4^	2.9
Alanine	4.0 × 10^−2^	1.1
Acetic acid	9.3 × 10^−11^	1.7
Hippuric acid	4.5 × 10^−3^	1.8
Meso-erythritol	2.1 × 10^−9^	1.5
Threonic acid	4.9 × 10^−8^	1.6
Butanoic acid	2.7 × 10^−2^	1.5
Inositol	8.9 × 10^−5^	1.1
Hydroxytryptophan	4.4 × 10^−5^	1.4
Methyllinosine	4.4 × 10^−5^	1.5
Xanthosine	2.4 × 10^−3^	1.3
Dimethylguanosine	1.9 × 10^−4^	1.8
Methylguanosine	2.0 × 10^−2^	1.1
Tryptophan	2.8 × 10^−3^	1.4

FDR, false discovery rate; VIP, variable importance into projection.

**Table 2:** The SE-estimated properties of DFO/TiO_2_ films versus different concentrations of DFO dye.

DFO	Energy Bandgap (eV)	Thickness (nm)	Refractive Index @550nm	Porosity, *p* (%)
10^−2^ M	2.62	356	1.624	69.4
10^−5^ M	2.97	386	1.592	71.32
0	2.95	300	1.514	75.85

**Table 3:** The mean fluorescence lifetime (amplitude weighted) of DFO in TiO_2_ thin films. λobs. = 450 nm; λex. = 370 nm.

C_DFO_ [M]	Mean Fluorescence Lifetime (ns)
2 × 10^−2^	0.39
2 × 10^−3^	0.51
10^−5^	0.63

## Supplementary Materials

The following are available online at https://www.mdpi.com/1996-1944/13/13/3014/s1, Figure S1: Negative control of the fluorescence micrograph without glycine, Figure S2: Fluorescence for 2 × 10^−2^ M with glycine, when the best fluorescence was obtained.
