# Supplementary material for: The Luminescence of 1,8-Diazafluoren-9-One/Titanium Dioxide Composite Thin Films for Optical Application
Source: Materials (Basel). 2020 Jul 6;13(13):3014. doi: 10.3390/ma13133014 (PMC7372385; doi:10.3390/ma13133014)
Supplement: Supplementary file 1 [file materials-13-03014-s001.pdf]

materials

Supplementary Materials

The Luminescence of 1,8-Diazafluoren-9-One/Titanium Dioxide Composite Thin Films for Optical Application

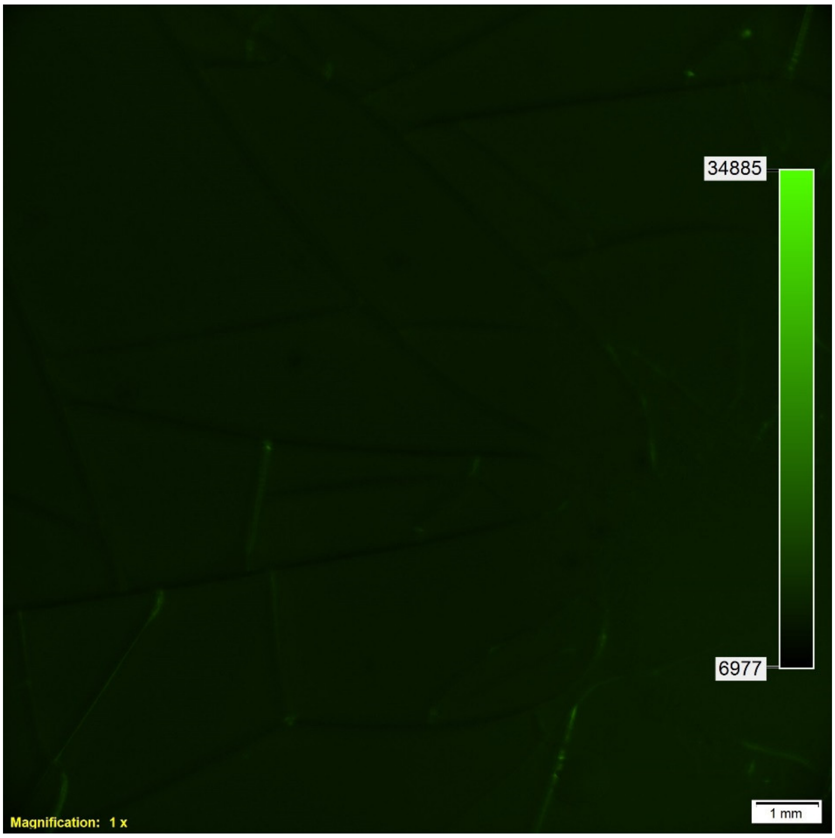

Figure S1. Negative control of the fluorescence micrograph without glycine.

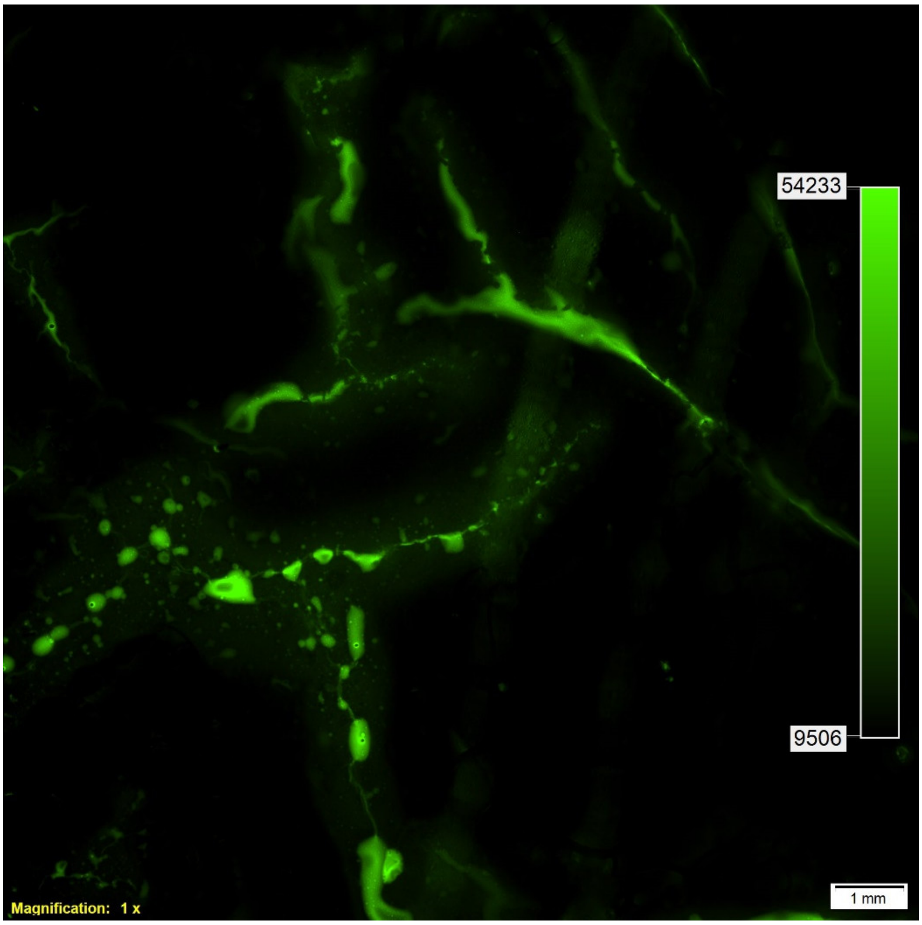

Figure S2. Fluorescence for 2x10-2M with glycine, when the best fluorescence was obtained.

© 2020 by the authors. Submitted for possible open access publication under the terms and conditions of the Creative Commons Attribution (CC BY) license (http://creativecommons.org/licenses/by/4.0/).
